# RITUALS OF INFANT DEATH: DEFINING LIFE AND ISLAMIC PERSONHOOD

**DOI:** 10.1111/bioe.12047

**Published:** 2013-08-02

**Authors:** ALISON SHAW

**Keywords:** personhood, pregnancy loss, stillbirth, British Pakistani Muslims

## Abstract

This article is about the recognition of personhood when death occurs in early life. Drawing from anthropological perspectives on personhood at the beginnings and ends of life, it examines the implications of competing religious and customary definitions of personhood for a small sample of young British Pakistani Muslim women who experienced miscarriage and stillbirth. It suggests that these women's concerns about the lack of recognition given to the personhood of their fetus or baby constitute a challenge to customary practices surrounding burial as a Muslim. The article suggests that these women's concerns cannot be adequately glossed as a clash of Islamic belief versus Western medicine. Rather, they represent a renegotiation of Islamic opinion and customary practices within the broader context of changes in the medical and social norms surrounding pregnancy loss and infant death in multi-ethnic British society.

## Introduction

At a prenatal appointment during her third pregnancy, a 23-year old British-born Pakistani Muslim woman asked if her doctors would provide ‘full management’ of this pregnancy. She was understandably worried because of her history. Her first baby, a boy, had been delivered by Caesarian section at 36 weeks after ultrasound scans had shown abnormalities. He had died three days later. Her second baby, a girl, had shown similarly fatal abnormalities on prenatal ultrasound but the doctors decided against a surgical delivery because of the associated medical risks. She had died in utero and was induced, stillborn, at 40 weeks. This time, Samina wished to remain in hospital for the remainder of her pregnancy. She also wanted an early surgical delivery ‘to get the baby out early, while it is still breathing, so I can make sure my father will be there to give *azān*, in case the baby stops breathing.’ The *azān* refers to the words of the Qalma (Muslim confession of faith) spoken into a newborn baby's ear. Samina's consultant explained that in this pregnancy there was no medical justification for keeping Samina in hospital or for a surgical delivery; all indications were that this would continue to be a normal pregnancy and result in a normal delivery. After Samina left the hospital, the genetic counsellor who had also been present during the consultation commented to me that Samina's desire for an early Caesarian section was ‘a question of religious beliefs up against Western medicine’.

Later that day, I found myself pondering the nurse's gloss of the incident as ‘religious beliefs up against Western medicine’. I had heard similar statements on a number of occasions during my observations of clinical encounters between health professionals and Pakistani Muslims patients. In one case the patient countered by stating his view was ‘personal, not religious’. Qualitative studies document this tendency for UK health care professionals to stereotype the situations of patients of ethnic minority backgrounds in terms of religion and culture, as if a non-UK ethnic heritage denotes a homogeneous, static entity of religion, culture and tradition.^1^ Such stereotyping can sometimes adversely influence access to healthcare. Evidence from a national confidential enquiry into screening for beta thalassaemia indicates, for example, that medical practitioners think there is no point in offering prenatal diagnosis to Muslim patients because Islam unconditionally forbids termination of pregnancy.^2^

Policy imperatives towards providing ‘culturally competent’ services aim to improve inequalities in access to health care by providing information about patients' cultural and religious backgrounds.^3^ However, simplified inventories of cultural and religious traits associated with particular minorities can reinforce the tendency to stereotype. Moreover, the drive towards cultural competency has also engendered uncertainty and disempowerment among professionals anxious not to cause offence or appear discriminatory when responding to the needs of patients whose ethnicities differ from their own.^4^ There are now calls for shifting away from cultural expertise models towards seeing patients as individuals who must negotiate across cultural and social contexts and the diverse religious and medical resources at their disposal.^5^

In this article, I offer an analysis of Samina's situation that seeks to demonstrate the limitations of glossing it as a case of ‘religious belief up against Western medicine’. I suggest that the key issue in understanding Samina's situation is the recognition of personhood in the context of changing meanings of pregnancy loss and infant death. I begin by situating my discussion with a brief review of anthropological approaches to personhood at the beginning and end of life. I follow this with a summary of current medical definitions and perceptions of pregnancy loss and infant death. After providing some details of the context and methodology of the research that informs this article, I then discuss how personhood at the beginning of life is constructed in the customary and religious understandings of my interviewees. I show how this is locally understood as insufficient for defining socially recognized Islamic personhood in cases of miscarriage and stillbirth. I suggest that the challenge this offers represents not so much a clash of Islamic belief versus Western medicine as a negotiation of Islamic opinions and customary practices within the context of recent changes in the medical and social norms associated with pregnancy loss and infant death in British society. Thus, Samina's situation reflects more broadly the social context in which pregnancy loss and infant death are now experienced and negotiated.

## Social Recognition at the Beginning and End of Life

When does a new human life begin? At what point does a new person come into being? And when do they die? A substantial anthropological literature shows the answers to these questions are not stable or self-evident. The biological events of birth and death do not unambiguously map onto their social recognition.^6^

Biological birth usually marks a transitional phase during which a baby is ritually prepared, through such procedures as bodily examination, washing, naming, baptism and the bestowing of inheritance rights, for being granted a social identity and being recognized as a member of a particular social collectivity. The ritual processes through which social personhood is bestowed are historically and socially variable, reflecting a society's beliefs, social structure and values.^7^ Personhood may be acquired incrementally across the life course, through childhood, puberty, marriage, parenthood and so on. Full personhood is in some societies denied to certain categories of person, such as women and children, and may be only fully achieved at death.^8^ The concept of social birth thus ‘highlights the gradual, malleable, and contested process through which personhood is often ascribed.’^9^

Moreover, the beginnings of this social recognition of a potential person frequently extend back in time to the ‘pre-birth’. This is the period of time between conception and the confirmatory social rituals conferring personhood.

At some point in the continuum of organic development, either before or after birth, a significant socio-moral identification takes place: what Marcel Mauss has called the ‘recognition’ of an individual child … ‘Recognition’ implies a pragmatic acceptance, conferring on the embryo, foetus, or infant at the least a provisional ‘personhood’, and an extension of basis physical care. This is not universal or automatic. Not all early human life is socially ‘recognized’ in this sense and partly as a consequence of the nature of such recognition not all survives.^10^

This period of pre-birth is marked by ambiguity. Historically, in England, fetal movement or ‘quickening’ was recognized as the standard by which a fetus' right to life was assessed in debates over abortion, but its timing was difficult to determine. Moreover, ‘provisional personhood’ is no guarantee of full personhood. Nonetheless, rituals to identify, protect and nurture the baby and pregnant mother usually accompany the ‘recognition of the child’. These rituals may invoke supernatural protection or entail ceremonial gifting, or provisioning of special foods and other treatments. Such preparation is not inevitable. It is partly a consequence of how the mother and people close to her envisage the future sociality of the child. Thus, the potential personhood of an illegitimate pregnancy or one destined for induced abortion may be played down, concealed rather than celebrated. And, as noted, the recognition of a child after birth does not necessarily lead to an immediate conferring of full personhood. Other conditions may need to be fulfilled, such as the absence of disability or death. Provisional or unconventional categories of personhood may be bestowed on live infants with disabilities, genital ambiguities, or where rates of infant mortality are high.

While rituals of birth are relatively ‘open, potentially plural and indeterminate’, rituals of death ‘lead to closure’.^11^ A corpse must be carefully handled and ritually prepared during the liminal phrase after biological death before the final ceremony.^12^ The loss of the person is then accomplished through rituals such as cremation and disposing of ashes, or burial and placing tombstones. The nature of the mourning and the degree of ritual elaboration of the commemorative ceremony are in general an indication of the status of the deceased among the living participants. For example, in the Andaman Islands, adults were mourned as a loss to the society, while the corpses of young children and infirm elderly people were disposed of with scant ceremony.^13^

Social death can precede biological death for persons whose physical bodies are alive but who have ceased to be recognized socially. This may take the form of early preparations for burial, burial alive, or leaving moribund individuals to die.^14^ In modern societies, social death may increasingly precede biological death, when very old, terminally ill or demented people no longer interact with or are recognized as persons by others.^15^ Increasingly, too, the process and timing of biological death is the medically managed outcome of complex negotiations between dying persons, their relatives and their doctors.^16^ Biological death may take place in stages, with ‘brain death’ occurring while life support equipment maintains a heartbeat and respiration.

## Pregnancy Loss and Infant Death

My interest here is in what happens when death intervenes during the ambiguous period after a ‘provisional’ child is recognized. What are the implications for the ‘recognition of the child’ when a pregnancy is ‘lost'? In contemporary medical discourse, an infant death is the death of a live-born baby, an early neonatal death occurring within the first seven days of birth, and a late neonatal death occurring between seven and 28 days. Pregnancy loss usually refers to miscarriages and stillbirths, which are considered pregnancies lost ‘from natural causes’ and are thus distinguished from pregnancies ‘ended’ by elective or therapeutic abortion.

Miscarriage, also known as spontaneous abortion, is the loss of an embryo or fetus and the commonest type of pregnancy loss. In most current biomedical definitions it occurs before around 24 weeks. A stillbirth is an inter-uterine fetal death, with a premature spontaneous or induced labour, which occurs after about 20–24 weeks. Arguably, pregnancies ended by means of induced abortion – that is, as selective or therapeutic rather than as spontaneous early or late abortions – are also pregnancies ‘lost’. Generally, though, miscarriage and stillbirth, medical abortion, and infant deaths are distinguished in biomedical discourse, with rather different implications for the management of the loss.

This distinction between miscarriage and stillbirth hinges on the concept of viability, when a fetus is considered able to survive independently. ‘The recognition of the child’ as a conditional person – the ‘viable fetus’ – now occurs at around 24 weeks of gestation in modern medical contexts, where viability is a central organizing principle in the management of pregnancy and birth.^17^ In practice, miscarriage can be difficult to distinguish from stillbirth, and has varying definitions and implications for parents. In England, Wales, and Scotland and, since 1995, the Republic of Ireland parents should register pregnancies lost after 24 weeks as stillbirths while in Australia this applies to pregnancies lost after 20 weeks. Miscarriage in these contexts is not the loss of a person in the way stillbirth is. However, the two are considered together in most pregnancy loss support groups.^18^

Where there is minimal or no prenatal screening, a fetus may be recognized as a person only late or ambiguously in pregnancy, usually at around the time that fetal movements are felt.^19^ Correspondingly, miscarriage and even stillbirth may not be recognized.^20^ In rural north India, for example, infant death is mourned only briefly and a lost pregnancy is ritually insignificant. An early miscarriage does not constitute the loss of a child because a child becomes ‘rooted’ in the womb only at three months after gestation, so any vaginal bleeding prior to this time is viewed as a delayed menstrual period and not a lost pregnancy.^21^

Whether a lack of social recognition of pregnancy loss results in unexpressed and unresolved grief is an open question. Rural north Indian women spoke without emotion about miscarriage and stillbirth, yet remembered with distress the deaths of toddlers and children.^22^ But the absence of expressed emotion does not necessarily indicate a lack of distress. If a miscarriage or stillbirth is not considered a baby or a person, and its loss is not ritually marked, women lack the opportunities ‘to mourn their loss openly or at length’^23^ but they may nonetheless experience grief. Elderly Northern Irishwomen remembered the emotional pain of miscarriage in the early 20th century, a time of high but declining rates of fertility, perinatal and maternal mortality. Their losses were not socially recognized at the time; ‘until the 1960s, unbaptized children were not usually buried in the consecrated ground of churchyards because the Catholic Church did not regard them as part of its community.’^24^

## Changing Meanings of Pregnancy Loss

The Northern Irishwomen in Roseanne Cecil's study were acutely aware that the meaning and management of miscarriage and stillbirth had changed dramatically over their lifetimes. Some of their premature babies might have survived had modern technologies of life support been available. The expanding repertoire of prenatal technologies is in turn closely linked to changes in perceptions of the status of the fetus.^25^

Until recently, it was standard practice for British hospitals to dispose of the bodies of miscarried and stillborn babies as clinical waste, rather than to offer spaces in memorial gardens. A ‘live’ birth was necessary for the loss to be socially recognized. Largely through the efforts of the pregnancy loss support movement,^26^ there is now recognition that miscarriage and stillbirth can bring deep long-lasting grief for parents, grief that may be lessened by socially acknowledging the loss. The pregnancy loss movement is ‘in effect, creating rites of separation for bereaved parents. Where in the past the remains were secreted away and disposed of by the hospital, many pregnancy loss support groups encourage parents to spend time with the child, holding it, bathing it, dressing it and to actively participate in a burial.’^27^ Parents are now offered professional emotional support and are encouraged to make their loss ‘real’ through the use of objects such as photographs and other memorabilia.^28^ The UK stillbirth and neonatal death charity (SANDS), founded in 1981, currently lists 51 memorial gardens for babies in England. Some of these gardens have names such as the ‘Rosebud baby garden’ and the ‘Forget-me-not baby garden’ but have only existed for about ten years.

It is in the context of these changing meanings of and practices for managing miscarriage and stillbirth that I return to my analysis of Samina's request for an early Caesarian in her third pregnancy. The evidence presented in this article will suggest that there remain striking differences in the ways in which individuals of diverse social backgrounds in Western societies interpret pregnancy loss and infant death, particularly where the status of the pregnancy or provisional person is uncertain, contested or ambiguously defined.

## Research Setting and Methodology

Pakistani Muslims constitute the second-largest ethnic minority population of South Asian origin in Britain and the largest ethnic subdivision of British Muslims. They are residentially concentrated in the Midlands and the North, in such cities as Bradford and Birmingham. Their settlement in Britain began after World War Two, as predominantly male labour migration. Wives and children joined men in Britain from the 1970s onwards. Subsequent new Pakistani immigration has mostly been through arranged transnational marriages, whereby spouses join second and third generation descendants of first generation migrants. Most British Pakistanis have ancestral and ongoing socio-economic connections in northern Panjab and Mirpur district in Azad Kashmir.

Rates of infant death vary by ethnic group in the UK and are highest among babies of Pakistani ancestry.^29^ Congenital anomaly is the main cause of infant mortality and morbidity among British Pakistanis and in this population is associated with the practice of consanguineous marriage, which confers an elevated risk of recessively inherited genetic conditions.^30^ Questions associated with the meaning and management of infant death for British Pakistani Muslims therefore have a particular salience in this context of elevated risk.

The cases presented in this article are taken from a fieldwork-based study in which, over four years, using methods of participant observation and interview, I discussed genetic risk and its management with 66 Pakistani Muslim couples referred to the genetic counselling service in High Wycombe, England.^31^ These referrals had been made following the diagnosis of a genetic condition in a pregnancy or child, or in the light of a family history of a genetic condition. Thirteen of the 66 couples had received a pre-natal or post-natal diagnosis of a fatal recessive condition affecting their fetus or baby and had between them experienced 19 pregnancy losses: two medical terminations, four stillbirths and thirteen neonatal infant deaths.

In this article, I draw from the narratives and reflections of six women in this subsample of 13 couples, using false names to preserve informants' confidentiality. These six women were all aged between 21 and 27, of Mirpuri heritage, and married to first cousins. Four of them were British born and educated, while two women were born and raised in Pakistan and had come to Britain following marriage. Interviews were conducted in English or in Urdu, in which I have a spoken competence.

In what follows, I discuss the constructions of personhood that shaped these women's experiences of pregnancy loss and infant death. In particular, stillbirth and its social recognition emerged as a central concern in the narratives of three young British-born women. I will suggest that these women's concerns represent a generational and contemporary cultural challenge to customary practices associated with pregnancy loss. This challenge needs to be understood against the broader backdrop of the recent pregnancy loss movement. I begin by presenting some details of the customary practices and beliefs associated with birth and death that my informants described. ‘Customary practice’, in my informants' usage, is not static, and combines elements of Islamic Sharia, locally understood Islamic practice, and Punjabi and Mirpuri culture. Disentangling religion from custom, or distinguishing a textual interpretation from the context in which it is customarily enacted, is not my concern here. Rather, I hope to demonstrate how elements of customary practice may be challenged and implicitly or explicitly renegotiated as the social context in which it is enacted changes.

## Protecting Pregnancy, Marking Personhood, Preserving New Life

For my informants, fetal movements in pregnancy were the clearest sign of new life. I was told that this is an indication that an angel has breathed *ruh* (life) into the baby. This is consistent with interpretations of the Quranic verses and Hadith (sayings of the Prophet) that indicate the transformation of a fetus into a living person occurs at 120 days from conception (17 weeks), when an angel breathes the *ruh* (spirit) into the fetus.

Protecting this new life was a key preoccupation for women who had experienced pregnancy loss or had an identified risk of abnormality in a pregnancy. I learnt about various *desi* (customary) and religious practices to promote a healthy pregnancy that might be used prior to conception – also as a remedy for infertility – and after a pregnancy is established. When I first interviewed Samina at her mother's house, Samina's Pakistan-born double first-cousin – these women's fathers are brothers and their mothers are sisters – was also present. Rubina had come to the UK after marrying Samina's brother. Both women had lost two pregnancies and were not yet pregnant again. Rubina's first baby, a girl born at 40 weeks and of a good weight, had died after six days. In her second pregnancy Rubina had felt the baby was no longer moving at the beginning of the seventh month and was sent for scans that showed the baby boy was dead. In the light of this history, Samina's parents were considering sending both women to Pakistan for a ‘rest’. They anticipated that this would include treatment in the form of herbal and homeopathic remedies, and massage given by a traditional midwife (*dāi*). The *dāi* would also advise special foods to promote and protect pregnancy. Samina's older sister and Samina's mother, who joined the interview part way through, also spoke about the value of prayer, particularly praying at the tombs of *pir*s (saints, or holy men) in Pakistan.

I should explain here that beliefs in the power of *pir*s to effect cures for a range of ailments are typically associated with South Asian Muslims of the Barelvi tradition. Such beliefs are characteristic of many Pakistani families from Azad Kashmir living in High Wycombe, and in other British cities such as Birmingham and Bradford, although they are also vigorously challenged as ‘customary’ by other, more orthodox Muslims. And accessing *pirs* does not necessarily require travel abroad. Samina had not been to Mirpur since her wedding, but in her second pregnancy she had worn a protective *tāviz*, prepared for her by a *pir* in Pakistan and sent to her by her mother-in-law. Rubina had returned to her parents in Pakistan during her second pregnancy and this visit had included praying at the tomb *ziārat* (tomb) of a *pir* in the locality of her parents' village in Mirpur.

When Samina became pregnant again, not long after our first interview, her family decided against the trip to Pakistan, considering it safer for Samina to avoid international travel and to have full medical surveillance of this pregnancy at the local hospital. As a compromise, Samina's mother took Samina and Rubina to a Bangladeshi *pir* in Birmingham, who provided them both with *tāviz* (amulets) containing words of the Qur'an. Samina showed me the three cloth amulets she was wearing – one round her neck, one on her abdomen, and one at the top of her calf, just below the knee. Samina's mother also wanted her daughter to have full access to biomedical expertise, and could not understand why, in the light of the reproductive history, Samina's doctors were not keeping her in hospital for the entire duration of the pregnancy. Yet the family also believed that all remedies, including Western medicine, had their limitations, and perceived God as the ultimate arbiter of successful or unsuccessful reproduction. As Samina's eldest sister said, with reference to Rubina's trip to Pakistan during her second pregnancy, ‘You can go to a *ziārat*. She went to a *ziārat*, but it still happened. It is her luck. And science, science cannot control everything. They [biomedical doctors] are not Gods. At the end of the day, we say it is in God's hands.’

After a baby is born, the first and, as discussed further below, arguably the most important marking of Islamic personhood is the *azān*. The baby may be named at this point but final decisions about a name may be made later, sometime after relatives in Pakistan have offered their suggestions. My interviewees mentioned several additional neonatal rituals performed during the early post-natal period to protect a vulnerable newborn baby from illness, evil spirits and even the malicious gaze of jealous neighbours. The *musalmāni* (circumcision) is a critical marker of Muslim maleness. For newborn babies of both sexes, head shaving is a purifying Punjabi ritual, newborn hair being *nāpāk* (impure) as a result of having been in the womb, in contact with the mother's blood. Parents or other relatives may also obtain *tāvi*s for babies and young children considered in need of supernatural protection.

## Rituals of Death

Just as God decides whether or not a baby will be born alive and healthy, He decides the moment of death; according to my interviewees, ‘Life and death is in God's hands.’ Muslim practice is to bury rather than cremate the dead. Indeed, my interviewees say, in the time between death and burial, the body and soul is especially vulnerable, and any bodily trauma is far more painful at this time than in life. Thus, post-mortems are not agreed to unless required by law, in which case in the UK the hospital will seek to return the corpse to the family for burial as soon as possible. For the same reason, Muslim burials should take place as soon after the death as possible, ideally in the ground where the person dies.

Preparation for a Muslim burial involves ritual washing of the body by close kin of the deceased or Muslim funerary services. Men wash a male corpse and women wash a female corpse. The body is then wrapped in white unstitched cloth in preparation for relatives and friends to pay their final respects before the burial. The body is then taken for *janāza*, the congregational funerary prayers, and to be laid in the ground, facing the *Ka'ba* in Mecca. Women may participate in *janāza* prayers if recited at home or at a mosque, but only men accompany the body to the burial ground. Afterwards, visitors usually come to the home of the deceased to offer condolences to the bereaved family. Funerals are usually attended by a wide range of people who travel from near and far to acknowledge the loss of one of their community.

Despite the Islamic recommendation that a person be interred quickly after death, it has been customary since the early years of Pakistani settlement in Britain for bodies of deceased adults to be returned to Pakistan. There, bodies are usually interred alongside graves of other members of their *birādari* (kinship group) in cemeteries located at the edges of migrants' villages of origin. First-generation migrants usually maintain it is important for relatives in Pakistan to view the deceased person and pay their final respects before burial. Moreover, relatives' prayers for the deceased are believed to accrue *sawāb* (blessing) to the deceased person's account with God, which will be weighed up on the Day of Judgment. It is thought that relatives are more likely to remember the deceased person and pray for them if their grave is nearby. The ideal place of burial, therefore, is near to the social body of the community, in a place that relatives will visit.

Increasingly British Pakistani Muslims are being buried in the UK, in cemeteries or sections of cemeteries allocated for Muslims following negotiations with local councils.^32^ Some Pakistan-born adults express a wish to be buried where their adult children are living, rather than in Pakistan. Sometimes, adult children of pioneer-generation migrants decide not to return their parents' bodies to Pakistan, so that they can more easily visit their parents' graves.^33^ However, the bodies of Pakistani Muslim children are nearly always buried in the UK rather than in Pakistan. In the Muslim section of the cemetery in High Wycombe, the Muslim graves are distinctive because the tombstones all face in the same direction, in neat rows. It is also striking that there is a roughly equivalent number of adult and of child graves, reflecting this history of burial practice. Some of these graves are unmarked grassy mounds of earth, not necessarily indicating neglect because a grave is in orthodox Islam supposed to be an unadorned, simply marked mound. Others, however, have elaborately carved tombstones, some procured from specialist stone-carvers in Mirpur and carrying inscriptions that offer insights into aspects of the migration and settlement history of this community.

## When Death Intervenes at the Beginning of Life

I now examine the implications of these customary ideas and practices surrounding the beginning and end of life for when death intervenes during the ambiguous phase of ‘conditional personhood.’ As we saw, quickening, or the breathing of *ruh* into a child, was a critical point in women's recognition of a potential child. This awareness of a child's life underpinned parents' concerns about the permissibility of facilitating death when considering medical abortion for fetal abnormality and when their consent was sought for the withdrawal of life-support from infants with fatal abnormalities. However, as I shall show in what follows, this definition of life was insufficient to guarantee the recognition of Islamic personhood for miscarried and stillborn babies.

Pakistan-born Sofia talked in some detail about her own experience of miscarriage. In Sofia's first pregnancy, at the 20-week scan doctors had detected fatal abnormalities associated with a known recessive condition and offered a medical abortion. The couple chose to continue with the pregnancy. Sofia had a surgical delivery at 38 weeks of a boy who died a few days later. He was named and buried in the Muslim cemetery. Sofia's next two pregnancies resulted in miscarriages at 10 and at 12 weeks.

Sofia told me that she did not recognize the first miscarriage as a miscarriage at the time: ‘It was like a heavy period, though there was something solid in what came out. I flushed it down the toilet. I did not know then that the doctors would have liked to look at it.’ Sofia was referring to a fact that, for her, was initially surprising: that doctors would have sent a blood or tissue simple for genetic analysis. In the next pregnancy, when her second miscarriage started, Sofia immediately contacted a doctor and was admitted to hospital.

Speaking in Urdu about these experiences, Sofia said that in each case she had ‘lost’ the pregnancy before she could feel a baby move. She expressed this loss in the Urdu idiom, ‘*hāmyla zāya hogayā*’, which literally means that the pregnancy ‘became wasted’. She also used the English word miscarriage in an alternative phrasing: ‘miscarriage *hogayā*’ meaning ‘miscarriage happened’. This phrasing signals that Sofia considered these to be lost pregnancies rather than lost babies, miscarriages rather than stillbirths.

The Urdu idiom for late miscarriage or stillbirth is ‘*bachchā gyr gayā*’, which means, literally ‘the baby fell’. *Bachchā* is the Urdu/Panjabi word for baby or child. A pregnancy lost after fetal movements are felt is therefore recognized as the loss of a child, whereas an early miscarriage remains a lost pregnancy, a loss of blood or tissue. This understanding of fetal development broadly parallels the Islamic belief that ‘life’ begins when the *ruh* (sprit) has been breathed into the baby. An authenticated Hadith describes a fetus as ‘semen’ at 40 days from conception, a ‘clot of blood’ at 80 days and a ‘lump of flesh’ at the 120-day point at which God sends an angel to breathe the *ruh* into the baby.^34^

There is also an important distinction between miscarriages and medically induced abortion in local Urdu and Panjabi idiom. Early or late miscarriages are events that happen to the mother, not events that the mother or another person ‘causes’ to happen. The translations of the Urdu expressions are that the pregnancy ‘becomes’ wasted or ‘a baby falls’. By contrast, medical abortions are events that involve human agency. The literal meaning of the sentences ‘*ws ne hāmyla zāya kar diya*’ and ‘*ws ne bachchā zāya kar diyā*’ are, respectively, ‘She [or he] *caused* the pregnancy [or the baby] to be wasted’ and, ‘She [or he] *caused* the baby [or the child] to be wasted’.

## Agency and Facilitating Death

This difference in expression also reflects an underlying moral distinction between spontaneous pregnancy loss and medical induced abortion. My interviewees expressed considerable disquiet over the idea of medical induced abortion even where fatal abnormality in the fetus had been confirmed by ultrasound scans or genetic tests. The dominant view was that abortion in such cases is murder, for which God will punish the murderer unless it is certain that the mother will die. Sofia recalled the option of abortion in her first pregnancy as an invitation to ‘waste’ the pregnancy, which she said, ‘our religion does not allow’. Later, in Sofia's fourth pregnancy, similarly fatal abnormalities were confirmed after the gestational cut-off for abortion. Sofia's doctors offered feticide [by injection] and an induced labour, but Sofia was clear that ‘I did not want to kill [the baby]’.

A consequence of the dominant view that a medical termination of pregnancy is sinful is that when abortion is carried out, it may need to be concealed from some family members. A couple might simply tell relatives that they ‘lost’ the baby, saying ‘the baby fell’ (*bachchā gyr gayā*), using the idiom for miscarriage. Occasionally women who choose to terminate an abnormal fetus without the support of a husband or in-laws may arrange for the procedure to take place in a hospital away from the their home town, to reduce the risk of malicious gossip or other harm. Older migrant-generation women tended to view the births of babies with disabilities as challenges sent by God, and which they should meet with patience (*sabar*), endurance and a faith in Allah's will. Locally, stories circulated about women who had terminations and were then ‘punished’ by Allah, for instance when their healthy children developed psychiatric or physical problems. The two women among my interviewees who chose abortion did so with considerable ambivalence and, later, strong feelings of wrongdoing.

British-raised Miriam expressed a personal view that medical abortion before 20 weeks might be acceptable. Miriam already had two healthy daughters when offered a medical abortion 20 weeks into her third pregnancy when scans showed a baby with fatal abnormalities. She considered but declined the abortion because she felt it was ‘too late’: ‘I might have gone for it in the early stages’ she said, but not after 20 weeks. She did not justify this view with reference to Islam.

British-raised Shakeela sought unsuccessfully to find any authoritative Islamic support to enable her to end a first pregnancy shown on ultrasound to have multiple fatal abnormalities. Following her mother's advice to trust in God, and informed by books stating that medical abortion is wrong for Muslims unless the mother's life is in danger, Shakeela decided against a termination. She proceeded to have a traumatic surgical delivery of a fatally malformed baby, who lived less than an hour, was named and buried in the Muslim cemetery.

At the baby's funeral, however, Shakeela learnt from a woman offering condolences that some Muslim scholars have issued authoritative alternative interpretations of the Islamic position on abortion. These interpretations include fetal abnormality as a reasonable ground for abortion before 120 days of conception, corresponding to about 19 weeks of the medical gestational calendar. *Fatwa*s to this effect have been issued in Kuwait, Saudi Arabia and Iran.^35^ In Pakistan, statements by Muslim scholars have reassured couples about using prenatal testing and abortion for thalassaemia.^36^ Abortion after 120 days is also permitted in most of these interpretations on grounds that include the expectation of fetal death or serious deformity.^37^ On learning this, Shakeela felt strongly that there should be more open discussion of Islamic opinions on abortion. Exercising agency in facilitating the death of a child was also a concern for four couples who consented to the withdrawal of life support from infants in special care. These couples told their families that ‘the baby died’ lest they be accused of permitting the killing of their babies.

## Stillbirth, Infant Death and Islamic Personhood

When I first interviewed Samina and Rubina they had each lost two pregnancies. Samina's first baby had died after 3 days; the second had died in utero and was induced at 40 weeks. Rubina's first baby had died after 6 days; the second had died in utero and was induced at 34 weeks. ‘The big ones’, Rubina told me in Urdu ‘are buried *upar* (above), while the little ones are buried *niche* (below)’. Rubina spoke of ‘the big ones’ by name, explaining that ‘in our religion’ a baby is only recognized by name if it has drawn breath and received *azān*. As I still looked puzzled over the distinction between burial *upar* and burial *niche*, Samina added some further explanation, in English:

The big ones are the two babies who lived a few days. They had drawn breath and so they had received the *azān* and could have *janāza*. They are in the Muslim section of the [local hillside] cemetery, at the top of the hill. The little ones [she signed as she said this] are the babies who were born dead; they are buried in the Snowdrop Garden [the memorial garden for miscarried and stillborn babies, located at the bottom of the hill at the entrance to the cemetery]. It is because they haven't had the *azān* … they can't do that if the baby does not breathe. If the baby breathes for a second, they can do it, but they have to do it quickly. If there is no one there [who can do the *azān*], the baby does not get the *azān* and cannot be buried in the Muslim cemetery.

The distinction implies two different kinds of personhood: a baby buried ‘above’ is recognized by name and as a Muslim, while the personhood of baby buried ‘below’ is almost completely denied. This difference in personhood is to some extent shown by how elaborately the graves ‘above’ and ‘below’ are marked. In the Muslim section of the cemetery, the graves of babies are clearly distinguishable from the adult graves by being smaller. Some of these small graves are simple grassy mounds, as are some of the adult graves. But many of them have elaborately decorated tombstones, some of them crafted by stone-carvers in Pakistan. These tombstones carry inscriptions that bear witness to the brevity of the child's life, noting a death that occurred a few moments, hours, days, or months of birth, or marking the short life of an older infant. These brief biographies of named infants convey profoundly a sense of the loss experienced by the parents.

In the Snowdrop Garden, by contrast, the Muslim graves are almost completely unmarked, although they are clearly recognizable because they are grouped together and all face the same direction. The non-Muslim graves are marked by flowers and inscribed tombstones while name plaques mark where ashes have been interred. A few of the Muslim graves still carry numbers that would enable the hospital or the parents to identify the baby, through the system used by the local National Health Service Trust to allocate spaces in the memorial garden. But these small mounds of earth are untended and overgrown with weeds, giving the impression that the Muslim parents of these babies do not visit. Six months after her stillbirth, Rubina told me that she had not been to the Snowdrop Garden, adding that ‘you can't visit except on special days’, although in fact the Snowdrop Garden is open to visitors at the normal opening times of the cemetery.

We can therefore interpret Samina's request for an early surgical delivery in her third pregnancy ‘to get the baby out early, while it is still breathing’ as a desire for her baby to have a fully recognized Islamic personhood. This recognized Islamic personhood is constructed locally through having a Muslim burial. A publicly acknowledged *janāza* is a crucial ritual marker of the infant's life, however brief, because it fully acknowledges the social and religious identity of the child. Moreover, it provides social recognition to the bereaved parents of the fact that they have lost a child, a recognition made for example in the fact that neighbours will come to the home to offer condolences after the funeral. Parents of miscarried or stillborn babies, or babies who die before receiving *azān*, are thus denied public recognition of both their child's personhood and their own bereavement.

Islamic personhood, as constructed in these accounts, is thus conditional upon the saying of *azān*, which in turn depends on a child drawing breath. Samina desired a surgical delivery so that these conditions could be met: ‘so I can make sure my father will be there to give *azān*, in case the baby stops breathing.’ My interviewees' view of *azān* as a prerequisite for naming and *janāza* reflects customary practice rather than Islamic orthodoxy. In orthodox Islamic scholarship, receiving *azān* is not obligatory prior to naming and *janāza*. According to Islamic scholarship, a baby must give some sign of post-natal life, like crying, breathing, or even silent suckling to receive *azān*. If a baby is born alive and dies within seconds then *azān* is unnecessary. It is also recommended that the baby's father, a senior male or an imam speaks the *azān*, but this is not a necessity and *azān* can, in principle, be spoken by anyone, including the baby's mother. This implies that the local practice of waiting for the arrival of a senior male relatives or an *imām* is also a customary, rather than a religious, requirement.

Two other young British-born women who had experienced stillbirths spoke openly about the distress they felt when their stillborn babies did not qualify for *azān*, naming, and *janāza*. Miriam who, as we saw, had declined an abortion, proceeded to a spontaneous labour and a normal delivery of a boy who had died in utero at 37 weeks. For Miriam, the tragedy of her son's stillbirth was that he was not formally recognized as a Muslim, because *janāza* could not be performed. Miriam and her Pakistan-born husband named their stillborn baby, and took photographs of him in the arms of his parents and sisters, so that they would remember him. She showed me the number card by which they can identify his grave in the Snowdrop Garden. Miriam said that she considered her son Muslim because he was born to Muslim parents. But in her wider family and community her son's personhood was not recognized:

If he had drawn even one breath, then we would have had a funeral, all the family would have been there, and he would have been buried in the Muslim section of the cemetery. But no, sometimes, it's like he never existed. One Pakistani couple, I heard, their baby was born dead, and they just left it in the hospital and went home.

While Miriam had the support of her husband in creating a private memorial for her stillborn son, this was not the case for Qudsia. When I spoke with her, Qudsia was in the process of separating from her Pakistan-raised husband and moving away from High Wycombe. The pregnancy had been her first, and fatal abnormalities indicating a recessive renal problem were detected by ultrasound scan. Qudsia decided to continue with the pregnancy. Her son was born prematurely at 33 weeks and because he did not breathe, even for a moment, he did not receive either *azān* or *janāza*. Qudsia spoke bitterly, with heartfelt emotion, expressing a combination of anger and grief, about the fact that her family did not recognize her baby as a person to be named:

My family does not understand it's a genetic problem. They blame me, for not drinking enough water during pregnancy. And because he was stillborn, my husband and parents would not recognize him as a person to be named because he had not drawn breath. They say he did not exist. So I named him myself. He is buried in the Snowdrop Garden. I went to his burial.

By naming her baby to acknowledge his post-mortem personhood, despite his not having drawn breath, Qudsia was defying the local customary burial practice. She was not, however, acting in defiance of Islamic orthodoxy, which distinguishes obligatory and recommended burial rites, and thus differs from local customary practice. According to Islamic scholarship, *janāza* can be done for a stillborn baby that has moved in the womb. It can also be done for a baby born alive who died before receiving *azān*. In all cases, naming the baby is recommended, again challenging the local, customary view. This inconsistency between local customary practice in cases of stillbirth and neonatal death and Islamic orthodoxy is consistent with interpretations of the Quranic verses and Hadith that indicate that the transformation of the fetus into a living person occurs at 120 days, when an angel breathes the *ruh* (spirit) into it the fetus.

## The Invisibility of Pregnancy Loss

Two of my interviewees commented that in some parts of rural Azad Kashmir and Northern Punjab – but not in urban areas – a miscarried or stillborn baby is buried not in the cemetery but outside the boundaries of the village, indicating a lack of social recognition for pregnancy loss. Miscarriage and stillbirth were, by and large, endured in silence, in my interviewees' narratives. Pakistan-born Sofia, speaking of her two miscarriages – the first of which she herself had scarcely recognized as such – commented on the lack of awareness of the pain of miscarriage in Pakistan, and the difficulties of talking about it, even in Britain:

Everyone knows about a child being born and then dying – because everyone knows when a child is born. But they don't know about the miscarriages. My mother [in Pakistan] told my husband that she had lost a child, when he visited her in Pakistan after our son had died. But he did not tell her about the miscarriages. It will just make her worried, and she is so far away. And I would not talk to my mother-in-law or sisters-in-law [in the UK] about it. You can't talk about it to people, because they make you feel that you are to blame. People say it is because you did this, or because you did that. Instead of giving you comfort and making you feel better, they make you feel worse, so it is better to say nothing at all.

Her mother, Sofia explained, would worry about the security of Sofia's marriage, because reproductive failure is customarily ground for a man to take another wife. This constituted a justified worry, since Sofia's husband's parents were indeed suggesting a divorce.

UK-born Miriam, whose son was stillborn, was more explicit about the emotional implications of pregnancy loss. She said she could only talk openly and she could weep with her husband, who wept too. But she could not share her grief with her father, or her mother, or even her sisters, to whom she felt otherwise very close. She told me she was reluctant to talk to the hospital's counsellor, because of normative pressure from her family and other Pakistani friends against admitting the need to:

They think it is means you are pathetic and they say they won't want it [bereavement counselling] … they completely conceal it [their grief] but it is an emotional and psychological burden. There is a lot of shame about these things. Our people should own up and admit that these things happen. Then when something goes wrong they don't talk about it and that is really sad. I ended up blaming myself for it [the stillbirth].

Miriam had heard of two other cases of stillbirth in the few months since her son was stillborn, but had not talked directly to the women concerned:

My cousin, I met her, and it was clear she did not want to say anything about it and I did not like to ask, it did not seem appropriate. But I wonder how she is feeling. The baby was not full term. It was born dead at six months. They did not say if it was a boy or girl and I did not ask. I presume it's in the Snowdrop Garden but I don't know for sure. I don't think she has talked to anyone in the family, though I think she will have talked to the hospital staff. She is just twenty-one. The other woman, she is older, she is a friend, or rather, a friend of a friend. I thought she'd had her baby as it was due a month before me and they noticed something at the scan too. She has taken it really badly. It was her first pregnancy and she had been married a long time. She lost the baby at six months, but I don't know exactly what was wrong or what happened, only that the baby died at six months … people don't open up. Her baby is in the Snowdrop Garden.

Miriam added that she felt particular sympathy for women who speak no English to whom this happens during their first pregnancy:

I can't see them getting any support. They just get on with it and hope the next one is fine. In Pakistan it is even harder because there are no scans, no facilities and it must be all hidden. It happens there too, of course, but no one opens up.

## Conclusion

Miscarriage and stillbirth were customarily invisible in these women's narratives of pregnancy loss. Women's narratives also identify ambiguity and concern over the customary determinants of Islamic personhood and entitlement to Muslim burial in cases of stillbirth and early neonatal death. New life was recognized at the point at which fetal movement is felt, a point consistent with interpretations of Islamic texts that acknowledge a transformation of the status of the fetus when the spirit (*ruh*) enters it. Locally, however, a ritually marked live birth was a necessary prerequisite for a Muslim burial. The anthropological literature has acknowledged the social and emotional significance of commemorating the dead. A funeral ‘may be considered to be the formal expression of the community's loss of one of its members. It affirms the worth of the deceased in the eyes of all who participate.’^38^ Moreover, its significance extends beyond the occasion of the ceremony itself to the living participants, acknowledging their loss and affirming their continuing worth within their community.^39^ The issue central to the cases discussed in this article was the acknowledgement of the loss within the local Muslim community, in particular in women's own extended families and social networks.

Within Islamic scholarship, there is religious ground for giving a stillborn baby of Muslim parents a Muslim funeral, for instance in the view that funeral prayers are recommended over a baby stillborn after four months gestation. The grounds for not doing so, as identified here, can therefore be interpreted as customary to a particular socio-economic context of Muslim practice, rather than embedded in Islamic text. These findings suggest a need for more information on these matters, as well as on the range of Islamic scholarship and legal opinion on abortion and end of life decisions.^40^ Women's concerns also imply that the customary practice in which miscarriage is rendered invisible and must be endured patiently is no longer appropriate in the contemporary British context. This implication was most explicit in the critical commentaries by British-born women, some of whom were challenging prevailing customary practice by adopting new practices of remembrance. This observation resonates with the observation that second and third generation British Pakistani women are challenging the ethic of patient, silent gendered suffering that prevails among first generation Pakistani women.^41^

The findings in this article are based on a very small number of cases of women all of specific ancestral origins in Mirpur. My analysis of customary practices associated with miscarriage and stillbirth may thus be limited in scope to this community and locality. It does not take into account other potential constraints on publicly acknowledged Muslim burial of infants, such as the cost of interment in the Muslim cemetery. This article analyses concerns about miscarriage and stillbirth among a small number of women who knew, from previous experience or from prenatal diagnosis, that there was a very real possibility their babies would die. I did not discuss miscarriage and stillbirth with a control group of Pakistani Muslim women whose pregnancies had been unproblematic, or who had no identified risk of genetic problems in children. The accounts discussed here were not elicited as part of a study of the ethno-physiology of pregnancy loss but emerged, quite unprompted, during interviews about genetic risk. Nonetheless, my indicative findings may be relevant in other British contexts, given the high rates of infant mortality and morbidity in the predominantly Mirpuri British Pakistani population. They also contribute more generally to understanding how women of diverse ethnic and social backgrounds are renegotiating the meaning and management of miscarriage and stillbirth in multi-ethnic Britain. I would conclude, then, that the vignette with which I introduced this article illustrates not a simple ‘clash’ of Islamic and ‘western values’ but contestations of customary definitions of life and Islamic personhood that offer insights into the changing social and cultural contexts in which pregnancy loss and infant death are experienced and negotiated.

## Notes

1 S. Chattoo, K. Atkin. 2012. ‘Race’ Ethnicity and Social Policy: Theoretical Concepts and Limitations of Current Approaches to Welfare. In Understanding ‘race’ and ethnicity in social welfare. G. Craig et al., eds. Bristol: Policy Press; L. Culley, N. Hudson. 2009. Commonalities, Differences and Possibilities: Culture and Infertility in British South Asian Communities. In Marginalized Reproduction: Ethnicity, Infertility and New Reproductive Technologies. L. Culley, N. Hudson & F. van Rooji, eds. London: Earthscan books.

2 B. Modell et al. Informed Choice in Genetic Screening for Thalassaemia during Pregnancy: Audit from a National Confidential Enquiry. BMJ 2000; 320: 337–341.

3 I. Papadopoulos. 2006. Transcultural Health and Social Care: the development of culturally competent practitioners. Oxford: Elsevier.

4 J. Kai et al. Professional Uncertainty and Disempowerment Responding to Diversity in Health Care: a Qualitative Study. Plos Med 2007; 4(11). http://www.plosmedicine.org.

5 Ibid; M.M. Lo, C.L. Stacey. Beyond Cultural Competence: Bourdieu, Patients and Clinical Encounters. Sociol Health Illn 2008; 30(5): 741–755.

6 S.R. Kaufman, L.M. Morgan. The Anthropology of the Beginnings and Ends of Life. Annual Review of Anthropology 2005; 34: 317–341.

7 M. Mauss. 1985 [1938]. A Category of the Human Mind: the Notion of Person; the Notion of Self. Reprinted in The category of the person: anthropology, philosophy, history. M. Carrithers, S. Collins & S. Lukes, eds. Cambridge: Cambridge University press; M. Carrithers, S. Collins & S. Lukes, eds. 1985. The category of the person: anthropology, philosophy, history. Cambridge: Cambridge University Press.

8 J.S. La Fontaine. 1985. Person and Individual: Some Reflections. In The Category of the Person. M. Carrithers, S. Collins & S. Lukes, eds. Cambridge: Cambridge University Press.

9 Kaufman and Morgan, *op. cit*. note 6, p. 321.

10 W. James. Placing the Unborn: On the Social Recognition of New Life. Anthropol Med 2000; 7: 168–189, p. 170.

11 James, *op. cit.* note 10, p. 184.

12 R. Hertz. 1960. [1907]. A Contribution to the Collective Representation of Death. Reprinted in Death and the Right Hand. R. Hertz. Aberdeen: The University Press.

13 A.R. Radcliffe-Brown. 1933. The Andaman Islanders. Cambridge: Cambridge University Press.

14 Hertz, *op. cit.* note 12; J. Goody. 1962. Death, property and the ancestors: a study of the mortuary customs of the Lodagaa of West Africa. London: Tavistock.

15 M. Mulkay. 1993. Social Death in Britain. In The Sociology of death: theory, culture, practice. Sociological Review Monograph 40. D. Clark, ed. Oxford: Blackwell; B.G. Glaser, A.L. Strauss. 1966. Awareness of Dying. London: Widenfeld and Nicholson; H. Sweeting, M. Gilhooly. Dementia and the Phenomenon of Social Death. Sociology of health and illness 1997; 19(1): 93–117.

16 Kaufman and Morgan, *op. cit.* note 6.

17 A. Christoffersen-Deb. Viability: a Cultural Calculus of Personhood at the Beginnings of Life. Medical Anthropol Q 2012; 26(4): 575–594.

18 L. Layne. Motherhood Lost. Women Health 1990; 16(3–4): 69–98.

19 James, *op. cit.* note 10.

20 R. Cecil. 1996. Introduction: an Insignificant Event? Literary and Anthropological Perspectives on Pregnancy Loss. In The anthropology of pregnancy loss: comparative studies in miscarriage, stillbirth and neonatal death. R. Cecil, ed. Oxford: Berg: 1–14; Layne, *op. cit*, note 18, p. 89.

21 P. Jeffery, R. Jeffery. 1996. Delayed Periods and Falling Babies: The Ethnophysiology and Politics of Pregnancy Loss in Rural North India. In The Anthropology of Pregnancy Loss: Comparative Studies in Miscarriage, Stillbirth and Neonatal Death. R. Cecil, ed. Oxford: Berg: 17–37.

22 Ibid.

23 Ibid: 29.

24 R. Cecil. 1996. Memories of Pregnancy Loss: Recollections of Elderly Women in Northern Ireland. In The anthropology of pregnancy loss: comparative studies in miscarriage, stillbirth and neonatal death. R. Cecil, ed. Oxford: Berg: 177–195, p. 181.

25 Ibid; Christoffersen-Deb, *op. cit.* note 17.

26 L. Layne. Pregnancy and Infant Loss Support: a New, Feminist, American Patient Movement? Soc Sci Med 2006; 62: 602–613.

27 Layne, *op. cit.* note 18, p. 76.

28 L. Layne. ‘He Was a Real Baby with Real Things’: a Material Culture Analysis of Personhood, Parenthood and Pregnancy Loss. Journal of Material Culture 2000; 5: 321–345.

29 Office for National Statistics. 2012. *Gestation-Specific Infant Mortality in England and Wales, 2010*. http://www.ons.gov.uk/ons/rel/child-health/gestation-specific-mortality-in-england-and-wales/2010/index.html. Accessed 28 February 2013.

30 S. Bundey, H. Alam. A Five-year Prospective Study of the Health of Children in Different Ethnic Groups, with Particular Reference to the Effect of Inbreeding. Eur J Hum Genet 1993; 1: 206–219.

31 A. Shaw. 2009. Negotiating risk: British Pakistani experiences of genetics. Oxford: Berghahn Books; A. Shaw. Risk and Reproductive Decisions: British Pakistani Couples' Responses to Genetic Counseling. Soc Sci Med 2011; 73: 111–120.

32 J. Eade. 1996. Nationalism, Community and the Islamisation of Space in London. In Making Muslim Space in North America and Europe. B. Metcalf, ed. Berkeley: University of California Press: 217–234.

33 A. Shaw. 2004. British Pakistani elderly without Children: an Invisible Minority. In Ageing without children: European and Asian Perspectives on elderly access to support networks. P. Kreager & E. Shroeder-Butterfill, eds. Oxford and New York: Berghahn Books: 198–222.

34 S. Shaikh. 2003. Family Planning, Contraception, and Abortion in Islam: Undertaking *khilafah*. In Sacred Rights: the case for contraception and abortion in world religions. D.C. Maguire, ed. Oxford: Oxford University Press: 102–128, p. 120.

35 M.A.S. Abel Haleem. 1993. Medical Ethics in Islam. In Choices and Decisions in Health Care. A. Grubb, ed. Chichester UK: John Wiley and Sons: 1–20; F.S. Alkurya, R.A. Kilani. Attitudes of Saudi families Affected with Haemoglobinopathies towards Prenatal Screening and Abortion and the Influence of Religious Ruling (fatwa). Prenat Diagn 2002; 21: 448–451; Shaikh, *op. cit*. note 34.

36 S. Ahmed et al. Prenatal Diagnosis of Beta-thalassaemia in Pakistan: Experience in a Muslim country. Prenat Diagn 2000; 20: 378–383.

37 V. Rispler-Chaim. The Right Not to be Born: Abortion of the Disadvantaged Fetus in Contemporary Fatwas. The Muslim World 1999; 89(2): 130–143; A.R. Omran. 1992. Family Planning in the legacy of Islam. London: Routledge; A.I. Al Aqeel. Islamic Ethical Framework for Research into and Prevention of Genetic Diseases. Nat Genet 2007; 39(11): 1293–1296.

38 Cecil, *op. cit.* note 24, p. 187.

39 P. Connerton. 1989. How societies remember. Cambridge: Cambridge University Press: 44.

40 A. Shaw. ‘They say Islam has a solution for everything, so why are there no guidelines for this?’ Ethical Dilemmas Associated with the Births and Deaths of Infants with Fatal Abnormalities from a Small Sample of Pakistani Muslim Couples in Britain. *Bioethics* 2011; doi:10.1111/j.1467-8519.2011.01883.x.

41 K. Qureshi, *Sabar*: Body Politics among Middle-aged Migrant Pakistani women. J R Anthropol Inst 2013; 19(1): 120–123. DOI: 10.1111/1467-9655.12006; see also K. Qureshi, K. Charsley, A. Shaw. Marital Instability among British Pakistanis: Transnationality, Conjugalities and Islam. Ethnic Racial Stud 2012; DOI: 10.1080/01419870.2012.720691.

